# Repeated Feedback Can Benefit Seven-Year-old’s Uncertainty Monitoring in a Memory Task

**DOI:** 10.1007/s41465-025-00322-8

**Published:** 2025-04-03

**Authors:** Florian J. Buehler, Simona Ghetti, Claudia M. Roebers

**Affiliations:** 1 University of Bern, Bern, Switzerland; 2 University of California Davis, Davis, CA USA

**Keywords:** Uncertainty monitoring, Metacognitive feedback, Performance feedback, Active control group, Training, Primary school children

## Abstract

Children’s ability to accurately monitor their performance is crucial for self-regulated learning and academic achievement, but interventions are rare. We aimed to improve young primary school childrens' (*N* = 127; *M* = 7.45 years) uncertainty monitoring with feedback. Participants attended six training sessions in which they received either metacognitive feedback, performance feedback or were assigned to an active control group. In the metacognitive feedback group, children received feedback on the correspondence between their accuracy in a memory task and their uncertainty monitoring (confidence judgments). In the performance feedback group, children received solely feedback on their accuracy in a memory task. In the active control group, children solved attention control tasks. Pre- and post-training, we assessed participants’ memory performance in a recognition test and uncertainty monitoring with confidence judgments. Results revealed that metacognitive, but not performance feedback, improved children’s uncertainty monitoring from pre- to posttest. However, metacognitive and performance feedback did not improve memory accuracy. The number of required sessions and items per session might be one of the most important aspects to be clarified in future studies.

**Supplementary Information:**

The online version contains supplementary material available at 10.1007/s41465-025-00322-8.

## Introduction

Children’s ability to accurately monitor their uncertainty is crucial for their self-regulated learning and academic achievement (Dunlosky & Metcalfe, 2009; Freeman et al., 2017; Schraw et al., 2006). In the context of memory decisions, uncertainty monitoring is the ability to introspect and evaluate one’s memory and includes experiencing higher confidence for correct than incorrect memories (Nelson & Narens, 1990). This is critical for recognizing errors and provides the basis for self-regulatory processes, such as allocating study time, selecting an answer, or asking for help (Destan et al., 2014; Hembacher & Ghetti, 2013). Although already 4-year-olds report higher confidence in correct than incorrect memories (Geurten & Willems, 2016; Hembacher & Ghetti, 2014), there is still much room for developmental improvements. For instance, also 6-year-olds tend to overestimate their performance (Destan & Roebers, 2015; Finn & Metcalfe, 2014) and do not attend to all the necessary cues (e.g., retrieval fluency) to provide calibrated confidence assessments (Koriat & Ackerman, 2010). Calibrated confidence indicates that children accurately relate their confidence to memory performance and do not overrate their memory accuracy. Therefore, training uncertainty monitoring may be beneficial for potentiating the building blocks of lifelong learning. However, interventions targeting children’s uncertainty monitoring are scarce. Thus, the present study compares two training protocols involving different types of feedback to an active control condition.

Improving children’s uncertainty monitoring requires understanding the mechanisms underlying constraints in uncertainty monitoring. Previous research shows that kindergarten and primary school children do not efficiently use all relevant cues (e.g., retrieval fluency, task difficulty) to inform their confidence assessments. For instance, 5- to 6-year-olds do not consider their past task performance when confronted with the same task again (Lipko et al., 2009, 2012). Other studies with 4- to 8-year-olds show that even when considering task difficulty, children overestimate their memory (Destan et al., 2014; van Loon et al., 2017). Children have difficulty relying consistently on valid cues and using them effectively for uncertainty monitoring. Thereby, feedback might help guide children’s attention and cognitive resources toward valid cues for uncertainty monitoring and encourage them to effectively use those cues.

### How to Encourage Uncertainty Monitoring Through Feedback

Feedback may generally benefit performance because it can help children recognize the essential features of a cognitive task, including sources of potential difficulty and error (Destan et al., 2014; Muis et al., 2015; van Loon & Roebers, 2020). Previous studies have concentrated on metacognitive and performance feedback regarding uncertainty monitoring. Metacognitive feedback informs children about the correspondence between their subjective confidence and actual monitoring accuracy (Geurten & Meulemans, 2017; van Loon & Roebers, 2020). Performance feedback informs children about their task accuracy (O’Leary & Sloutsky, 2017; Oudman et al., 2022; van Loon & Roebers, 2017, 2020; van Loon et al., 2017). Each approach has advantages and disadvantages, which we will review next.

Van Loon and Roebers (2020) compared metacognitive feedback with performance feedback about an analogical reasoning task in 5- to 6-year-old kindergarten children. Compared to a control group receiving no feedback, children in the monitoring and performance feedback groups exhibited better uncertainty monitoring and detected more errors after three training sessions. Moreover, the metacognitive feedback group detected more errors than the performance feedback group. However, there was still much room for improvement, as even children in the metacognitive feedback group did not recognize two-thirds of their errors. In sum, metacognitive feedback might be more beneficial than performance feedback for children’s uncertainty monitoring because it might make children aware not only of their mistakes but also of their illusory feelings of confidence, perhaps promoting a more careful examination of informative cues. In line with these first findings, studies with adults suggest significant benefits of metacognitive feedback for monitoring accuracy and even higher accuracy on the memory tasks associated with the feedback (Callender et al., 2016; Miller & Geraci, 2011; Nietfeld et al., 2006). However, one study with adolescents did not find benefits of metacognitive feedback in a problem-solving task (Raaijmakers et al., 2019). Despite being a promising approach to improving uncertainty, research on metacognitive feedback is scarce.

A second approach focuses on providing feedback based on task performance. This approach is based on the idea that when children are confronted with trial-by-trial feedback on performance accuracy, they might learn to recognize and use mnemonic cues associated with correct or incorrect outcomes, resulting in increased calibration between accuracy and confidence (Efklides & Metallidou, 2020). In this context, three studies have reported that performance feedback decreased overconfidence in 6- to 10-year-old children and increased children’s error monitoring in a variety of tasks, including recognition memory, concept learning, and arithmetics (Oudman et al., 2022; van Loon & Roebers, 2017; van Loon et al., 2017). In these three studies, children indicated monitoring judgments twice: Firstly, after responding (no feedback) and secondly, after receiving performance feedback. This indicates that children relied on performance when they gave monitoring judgments for the second time. However, whether children benefit from performance feedback on trials for which they did not receive direct feedback remains unknown. Compared to the previously outlined studies (Oudman et al., 2022; van Loon & Roebers, 2017; van Loon et al., 2017), van Loon and Roebers (2020) provided performance feedback after 5- to 6-year-old children indicated their monitoring judgments and before the subsequent trial in an analogical reasoning task. Uncertainty monitoring and error recognition were higher in the performance feedback group compared to a group receiving no feedback, but children in the performance feedback group remained overconfident. Overall, results suggest that already 6-year-old children can benefit from performance feedback; however, they remain overconfident, leaving considerable room for further development. Additionally, performance feedback seems to increase children’s task performance.

Other studies have shown that performance feedback fails to reduce overconfidence in 4- to 5-year-old children in memory tasks, even after receiving feedback multiple times (Lipko et al., 2009, 2012; O’Leary & Sloutsky, 2017; Xia et al., 2022). For example, performance feedback did not decrease 5-year-olds’ overconfidence in a visual discrimination task (O’Leary & Sloutsky, 2017). In contrast, 8-year-old’s overconfidence declined across task repetitions (Lipko et al., 2012). This indicates that performance feedback might benefit more advanced primary school children but not necessarily preschool and kindergarten children. This may be because performance feedback alone is insufficient for young children to discover the most informative and valid cues associated with their accuracy and then translate those into more calibrated confidence ratings.

Moreover, it seems easier to benefit from performance feedback when feedback is provided trial by trial on monitoring judgments (Oudman et al., 2022; van Loon & Roebers, 2017; van Loon et al., 2017), compared to when performance feedback is provided after a block of trials on global monitoring judgments (i.e., performance postdictions) (Lipko et al., 2009, 2012; O’Leary & Sloutsky, 2017; van Loon & Roebers, 2020; Xia et al., 2022). Similarly, studies with adults reported that trial-by-trial performance feedback increased monitoring accuracy (Haddara & Rahnev, 2022), while global performance feedback (on the overall task performance) did not increase monitoring accuracy (Miller & Geraci, 2011). In sum, previous research suggests that performance feedback might be most beneficial for school-aged children when provided trial-by-trial-wise.

Overall, the literature review on metacognitive feedback and performance feedback reveals that studies are sparse, and findings are mixed. Critically, only one study with adolescents investigated the transfer effects of metacognitive feedback on an independent task (Raaijmakers et al., 2019). Moreover, none of the previous studies engaged children with repeated feedback opportunities over a longer time; instead, participants received feedback only once in a few trials (O’Leary & Sloutsky, 2017; Oudman et al., 2022; van Loon & Roebers, 2017, 2020; van Loon et al., 2017) or at the end of the task (Geurten & Meulemans, 2017; Lipko et al., 2009, 2012). Improving uncertainty monitoring likely requires multiple repetitions distributed over multiple sessions (van Loon & Roebers, 2021). More research on extensive experience with metacognitive and performance feedback is necessary to clarify the role of feedback in children’s uncertainty monitoring.

### The Present Research

The main goal of the present study was to compare the benefits of metacognitive feedback vs. performance feedback for first graders’ (7-year-olds) uncertainty monitoring. We targeted first graders because the transition to school is a crucial age window for metacognitive development (Roebers, 2017). While first metacognitive skills are already developed, there is still substantial potential for further improvements (Destan & Roebers, 2015), suggesting that interventions may be especially beneficial in this age. We were specifically interested in contrasting the effects of two training conditions, a metacognitive feedback and performance feedback condition, compared to an active control condition. Our training conditions build on van Loon and Roebers (2020), but we included multiple training sessions and a transfer task in the posttest. Critically, we investigated how these training conditions affected children’s uncertainty monitoring and accuracy on a memory task at posttest with stimuli different from the training sessions. In other words, we used the most conservative but robust approach to examining training effects. If improvements are observed from pre- to posttraining in a task different from that used during training, we can be more confident that the effects will transfer across tasks. This is crucial as previous research reveals limited cognitive skill transfer across tasks (Clerc et al., 2014). The present study is a critical step towards better understanding the mixed findings regarding the potential benefits of metacognitive and performance feedback or performance feedback alone.

Our training conditions were delivered across six sessions on a tablet. Previous research relied on a similar number of sessions and has shown that children (5- to 10-year-olds) have positive attitudes toward tablets and that computerized tasks are suitable for training children’s metacognition (Cornoldi et al., 2015; Macoun et al., 2022; Muis et al., 2015; Takacs & Kassai, 2019). The metacognitive and performance feedback groups did a memory task. The metacognitive feedback group received feedback on their task accuracy and additional feedback about the accuracy of their confidence judgment. The performance feedback group received feedback on their task accuracy but no feedback on the accuracy of their confidence judgments. The active control group did a simple multi-trial attention control task. The attention control task was briefly interrupted when they committed an error (performance feedback), but participants did not provide confidence judgments. The comparison to an active control allows us to ensure that any metacognitive or performance feedback effects are not due to generic and unspecific forms of feedback and extraneous variables associated with repeated contact with the experimenter or participants’ expectations about improvements after repeated testing (e.g., placebo effect) (Green et al., 2019). We also included measurements of individual differences, which have been shown to be related to uncertainty monitoring, such as basic cognitive abilities (i.e., executive functions, working memory, fluid intelligence, receptive grammar) (Buehler et al., 2025; Gonzales et al., 2022; Krebs & Roebers, 2012; Roebers, 2017), and home environment (i.e., parental education) (Carr et al., 1989). These measurements allow us to isolate training effects from cognitive and environmental variables.

We made several predictions. We predicted that uncertainty monitoring abilities in the metacognitive and the performance feedback groups would increase from the pre- to posttest compared to the active control group. Furthermore, we expected metacognitive feedback to be more beneficial than performance feedback (van Loon & Roebers, 2020). Although our study focused primarily on training effects on uncertainty monitoring, we also explored whether there would be training effects on memory accuracy (Callender et al., 2016; Muis et al., 2015; Nietfeld et al., 2006; van Loon & Roebers, 2020). To assess children’s uncertainty monitoring, we computed the mean difference between confidence judgments for correct and incorrect recognition memory decisions (Dunlosky et al., 2016; Schraw, 2009), consistent with several studies on metamemory development (Bayard et al., 2021; Fandakova et al., 2017; Hembacher & Ghetti, 2014).

## Materials and Methods

### Participants

We based our sample size on a power analysis using G*Power 3.1.9.7 software (Faul et al., 2007) for a 2 (Time: pre- vs. posttraining) × 3 (condition: metacognitive feedback vs. performance feedback vs. active control) mixed ANOVA. Based on the scarce previous findings on the benefits of feedback for children’s metacognition (e.g., O’Leary & Sloutsky, 2017; van Loon & Roebers, 2020), we assumed a conservative effect size of Cohen’s *d* = 0.25, an alpha level of 0.05, a desired power of 0.80, and a correlation between repeated measures of 0.5. This resulted in a sample size of 147 participants, which we rounded to a target sample size of 150 participants (50 per group). As more children per classroom participated than expected, our sample size was slightly higher. We recruited 183 participants from public schools in regular classrooms near a mid-sized Swiss town. As preregistered, we excluded children who scored below chance level (< 25%; *n* = 46) and children who correctly recognized more than 75% (*n* = 10) of the items during the pretest uncertainty monitoring task. This exclusion was necessary to ensure that all children could complete the task but also exhibited “enough” incorrect answers, which is crucial for measuring uncertainty monitoring. The final sample consisted of 127 participants (age = 7.45 years; SD = 0.46 years; range = 6.33–8.67 years; 54% male). The mother tongue of most children was German (66%). We assessed parents’ highest education as a measure of socioeconomic status: 6% finished obligatory school, 24% had vocational training, 22% had a high school degree, 29% had a university degree, and 18% chose not to report their highest educational degree. This is comparable to the region’s average education level (Federal Statistical Office, 2021).

### Procedure

We recruited participants through local school districts in Switzerland. Once school districts agreed to participate in our study, we contacted individual teachers within the districts, who assisted with informing families of the upcoming research. Parents and guardians were informed about the study but blinded about the condition their children were assigned to. Children whose parents or guardians gave written consent to participate were included (183 out of 206 contacted parents provided consent). Children also verbally agreed to participate. The ethics committe for the Faculty of Humanities at the University of Bern approved the study (approval number: 2020–10-00005).

We tested children in groups (up to 20) in their usual classroom setting. All tasks were fully computerized and conducted on tablets (Samsung Galaxy Tab S4 and Samsung Galaxy Tab A7) with a touch screen (10.4″ and 10.5″). The task instructions were given auditorily via headphones. For technical support and questions, two to three trained experimenters assisted all testings (pre-/posttest and training sessions). We assessed children’s uncertainty monitoring at pre- and posttest. We also assessed individual difference measures known to be related to uncertainty monitoring, such as receptive grammar, executive functions, working memory, and fluid intelligence (Buehler et al., 2025; Gonzales et al., 2022; Krebs & Roebers, 2012; Roebers, 2017). Between the pre- and posttest, participants completed six training sessions. Pre- and posttests lasted approximately 60 min (divided into two sessions to avoid fatigue), and the training sessions were 15 min each. We assigned participants’ classrooms randomly to one of the training groups: metacognitive feedback (*n* = 51; corresponding to 4 classrooms), performance feedback (*n* = 33, corresponding to 3 classrooms), and active control (*n* = 43, corresponding to 4 classrooms). Participants and teachers were blinded to the assigned condition. Additionally, since classrooms were from different schools, participants had no opportunity to interact with participants from other classrooms or to gain information about other conditions. Blinding experimenters to the conditions was not possible. However, the fully computerized assessments and interventions minimized potential experimenter biases and ensured a highly standardized procedure.

### Pre- and Posttest

**Fig. 1:**
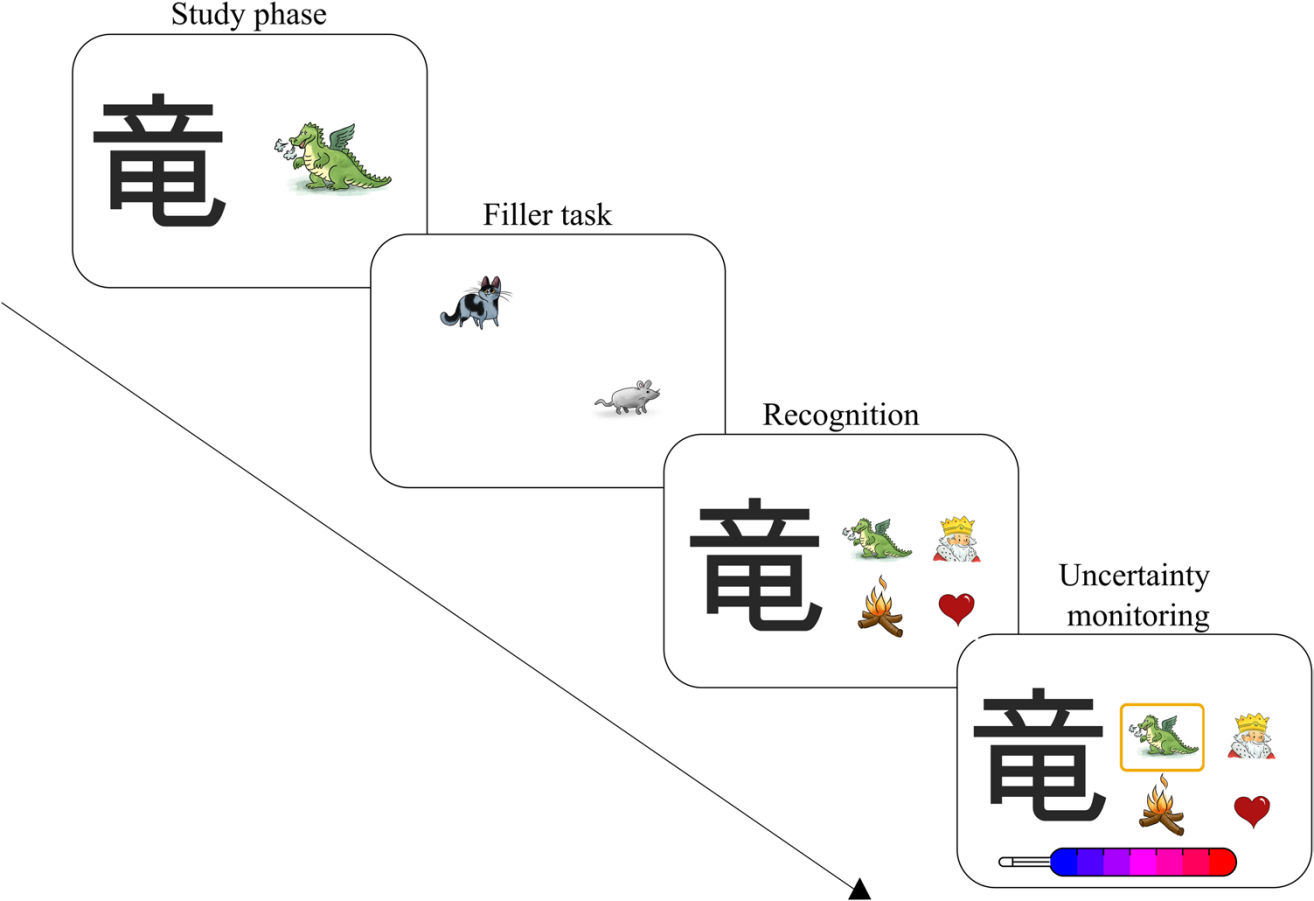
Pretest and posttest uncertainty monitoring. *Note. *Study phase: learning 16 Kanji-picture pairs; Filler task: 1 min. mouse-catching game; Recognition: selecting the corresponding picture pairs out of 4 options; Uncertainty Monitoring: indicating Confidence Judgments for each recognition selection

We assessed pre- and posttest uncertainty monitoring in a paired-associates recognition memory task. Similar tasks were used in our previous studies (e.g., Bayard et al., 2021; Destan et al., 2014). The task consisted of 16 pairs of pictures, each including a Japanese Kanji symbol and a picture representing its referent. We had two task versions, so participants learned different pairs of pictures in pre- and posttests. The order of the versions was counterbalanced across subjects. The task difficulty of the two versions was similar (version A = 46%; version B = 46%). The task instructions were computerized and included a practice trial to familiarize the participants with the touch screen, the recognition test, and the confidence scale. Only participants who successfully solved the practice trial could advance to the actual task. Participants who made a mistake in the practice trial received an additional face-to-face explanation from the experimenter supervising the children. The uncertainty monitoring task was divided into three phases: study phase, recognition test, and uncertainty monitoring (see Fig. 1).

In the study phase, participants were told to remember each of the 16 pairs of pictures. The pairs were presented in random order. Each pair was shown for 5 s. We piloted a large pool of item pairs beforehand to ensure sufficient variability concerning item difficulty. In the study phase, we included pairs with a difficulty index between 0.11 and 0.78. After studying the 16 picture pairs, participants executed a filler task (1 min) to prevent rehearsal. In the filler task, the participants steered a cat with one finger and tried to catch a mouse.

In the recognition test, the participants saw one Kanji at a time and had to choose the corresponding picture out of four alternative pictures (Fig. 1). The participants were familiar with all distractors because they had all been presented during the study time. The distractors were a combination of target pictures for different Kanjis. The distractors were randomized, but the randomization was constrained, so each picture was equally often shown as a distractor (two or three times per participant). Participants should choose one of the four pictures by double-clicking to continue the task. The requirement for a double-click allowed participants to change their choices if they wanted. As a measure of memory accuracy, we computed the mean percentage of correctly recognized Kanjis out of the 16 to-be-remembered pairs for each participant. For recognition, Cronbach’s *α* at pretest (version A = 0.40; version B = 0.47) and posttest (version A = 0.69; version B = 0.49) indicated acceptable internal consistency.

For uncertainty monitoring, participants indicated how confident they felt about each recognition decision immediately after selecting an answer by using a 7-point Likert scale—presented as a thermometer—ranging from very uncertain (blue, coded as 1) to very certain (red coded as 7) adapted from Koriat and Shitzer-Reichert (2002). Participants had to double-click to confirm their confidence judgment. To compute uncertainty monitoring, we subtracted the mean confidence judgments for incorrectly recognized Kanjis from mean confidence judgments for correctly recognized Kanjis (cf. Dunlosky & Thiede, 2013; Roebers, 2002). Positive values indicate that participants are more confident in correct than incorrect memories. The difference score is relevant to investigating training effects, as feedback aims to decrease confidence in incorrect memories and increase confidence in correct memories (see Table 1 for feedback). For confidence judgments, Cronbach’s *α* at pretest (version A = 0.93; version B = 0.94) and posttest (version A = 0.90; version B = 0.90) indicated a high internal consistency in the tendency to report higher or lower confidence judgments.

**Table 1:** Auditive metacognitive and performance feedback

Recognition	Confidence judgments	
	**Very uncertain/uncertain**	**Certain/very certain**
*Metacognitive feedback*		
**Correct**	Yes, that is the right food. But it is too bad that you were *(very) uncertain* about your answer	Yes, that is the right food. It is good that you were (*very) certain* about your answer
**Incorrect**	Oh no, that is not the right food. Do not worry it is a difficult task. But it is good that you were (*very) uncertain* about your answer	Oh no, that is not the right food. Don’t worry it is a difficult task. But it is too bad that you were *(very) certain* about your answer
*Performance feedback*		
**Correct**	Yes, that is the right food	
**Incorrect**	Oh no, that is not the right food. Do not worry, it is a difficult task	

*Note.* After each Confidence Judgment in the uncertainty monitoring phase, participants received metacognitive feedback or performance feedback via headphones. The table shows metacognitive and performance feedback based on recognition performance and Confidence Judgements

### Assessments of Individual Differences

We assessed individual differences with measurements of parental education, receptive grammar, executive functions, working memory, and fluid intelligence.

#### Parental Education

We asked parents in a questionnaire to indicate their highest education level: 0 = *no school education*; 1 = *obligatory school*; 2 = *vocational training*; 3 = *High School*; 4 = *University*. We relied on the highest reported score by one of the parents.

#### Receptive Grammar

We assessed receptive grammar with a computerized version of the TROG-D (Fox-Boyer, 2011). Participants heard sentences via headphones and had to choose a corresponding picture out of four alternatives. The TROG-D includes 21 blocks with four items each. To ensure children understood the task, they had to pass the first block (four items) as a practice trial and received additional support from an experimenter if they did not. The task ended after five consecutive blocks with at least one incorrectly solved item. We computed the sum score of correct blocks (all four items correct) per participant. Possible scores range from 0 to 20. Internal consistency was good (Cronbach’s *α* = 0.86).

#### Executive Functions

We assessed inhibition and shifting with the frequently used Heart and Flower task (Camerota et al., 2019; Davidson et al., 2006a; Roebers, 2022). Participants reacted to a heart or a flower presented on the screen’s left or right side by pressing external buttons. For hearts, participants had to press the button on the same side as the presented heart. For flowers, the participants had to press the button on the opposite side as the presented flower. The task consisted of three blocks: (1) In the congruent block, only Hearts were presented (24 trials). (2) In the incongruent block, only Flowers were presented (36 trials). (3) In the Mixed block, Hearts and Flowers were presented (60 trials; every fourth to sixth trial was a flower). To ensure children understood the task, they had to pass a practice trial and received additional support from an experimenter if they did not.

For inhibition, we computed the mean score of correct trials and the mean reaction time of correct trials in the Flower block per subject. Internal consistency was excellent for correctness of trials (*α* = 0.97) and good for reaction time per trial (standardized *α* = 0.79). For shifting, we computed the mean reaction time of correct trials and the mean reaction time for correct trials in the Mixed block per subject. Internal consistency was excellent for correctness of trials (*α* = 0.99) and good for reaction time per trial (standardized *α* = 0.83). Based on reaction times (RT), we excluded trials at the anticipatory level (RT < 250 ms), and trials with RTs higher than 2500 ms. This concerned overall 3.62% of the trials.

#### Working Memory

We assessed visuo-spatial working memory with a computerized Position Span task (Frick & Möhring, 2016) based on the Corsi-Block-Tapping Task (i.e., Corsi, 1972). Participants saw a mole (1200 ms) that appeared and disappeared in different locations on a 4 × 4 grid. Then they had to indicate the locations they had seen the mole in reverse order (see Maurer & Roebers, 2021). To ensure children understood the task, they had to pass a practice trial and received additional support from an experimenter if they did not. The task started with a sequence of six trials with two locations. If at least three out of six trials within a sequence were solved correctly, the number of locations increased by one in the next sequence. The task ended when more than three trials within a sequence were answered incorrectly. We relied on the total number of correctly remembered trials to measure working memory. Possible scores range from 0 to 36. Internal consistency was good (*α* = 0.81).

#### Fluid Intelligence

We measured fluid intelligence with a computerized version of the *Odd-Item-Out* subtest from the RIAS (Hagmann-von Arx & Grob, 2014; Reynolds & Kamphaus, 2003). Participants had to identify an incongruous stimulus in a set of related stimuli (matrices). To ensure children understood the task, they had to pass a practice trial and received additional support from an experimenter if they did not. The task ended after three consecutively incorrectly solved matrices. We computed a sum score per participant as a measure of fluid intelligence. Correct answers within 30 s were scored with 2 points, correct answers within 50 s were scored with one point, and answers exceeding 50 s were scored with 0 points. Possible scores range from 0 to 102. Internal consistency was high (*α* = 0.89).

### Training Sessions

Children were assigned to one of three training conditions. They either received metacognitive feedback or performance feedback or were assigned to the active control group.

#### Metacognitive Feedback

**Fig. 2:**
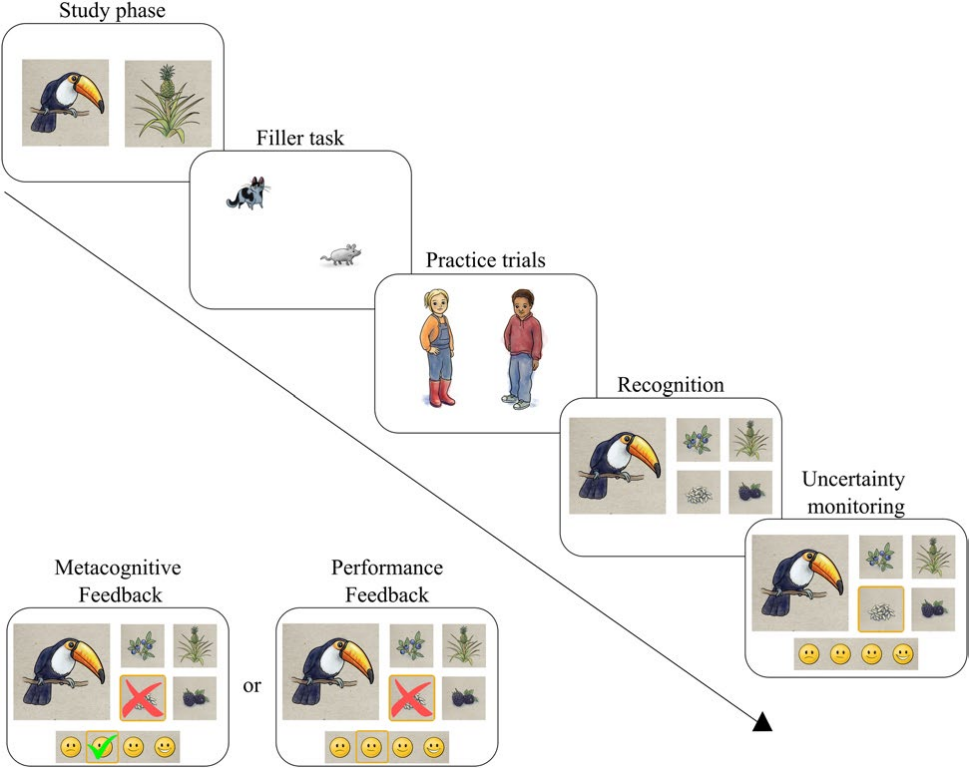
Metacognitive and performance feedback sessions. *Note*: Study phase: learning 12 animal-food pairs; Filler task: 1-min mouse-catching game; Practice trials: characters from the cover story solving four trials, Recognition: selecting the corresponding picture pairs out of 4 options; Uncertainty Monitoring: indicating confidence judgments for each recognition selection; Metacognitive Feedback: recognition and monitoring feedback; Performance Feedback: recognition feedback

We trained participants’ uncertainty monitoring in a paired-associates task, different from that used for the pre- and posttraining assessment. Specifically, participants were told to remember 12 pairs of animals and their preferred food. We had six topics—one for each appointment—including different pairs of animals and food (e.g., animals from the forest, fish, birds, gnawers, desert animals, and insects). The training task was divided into four phases: study phase, recognition test, uncertainty monitoring, and metacognitive feedback (see Fig. 2).

In the study phase, each animal-food pair was shown for 5 s. After studying the picture pairs, participants executed the same mouse-catching filler task (1 min) as in pre- and posttest to prevent rehearsal.

In the recognition test, participants chose the corresponding food out of four alternatives for each animal. The participants were familiar with the presented distractors as they were shown as target pictures for different animals in the learning phase. However, the presented distractors were not randomized and selected based on their perceptual similarity with the target. Participants were forced to choose one of the four pictures to continue the task. Participants had to double-click to select and confirm their answers.

For uncertainty monitoring, participants gave a confidence judgment immediately after selecting an answer in the recognition test. Participants had to indicate their confidence on a 4-point Likert scale—presented as smileys—representing *very uncertain*, *uncertain*,* certain*, and *very certain.* Participants had to double-click to select their confidence judgment. Children received elaborate explanations with examples and practice with feedback for ensuring the correct use of this 4-point scale at the beginning of the first training session (see below). We elected to use a different scale for training to prevent children from simply learning to map certain selections on the confidence scale and to ensure metacognitive transfer in the posttest from the training. Children understood and easily learned to use both the thermometer and the smiley scale.

Participants received metacognitive feedback after each confidence judgment in the uncertainty monitoring phase. The metacognitive feedback group received performance feedback and additional feedback on the correspondence between their performance and confidence judgment. Feedback was given visually (green tick for correct recognition and accurate monitoring judgments, red cross for incorrect recognition and inaccurate monitoring judgments) and auditive. Participants received positive feedback for correct recognition and (very) certain judgments and for incorrect recognition and (very) uncertain judgments. Participants received child-appropriate negative feedback for incorrect recognition and (very) certain judgments and correct recognition and (very) uncertain judgments*.* See Table 1 for auditive metacognitive feedback.

Notably, for practice purposes, two characters involved in the task’s cover story solved the first four trials of each training session (recognition, uncertainty monitoring, metacognitive feedback). The characters gave an example for each corresponding performance-monitoring combination (correct and very certain, correct and certain, incorrect and very uncertain, incorrect and uncertain). After these first four trials, participants responded to and received feedback on the remaining eight trials.

#### Performance Feedback

This training was identical to the metacognitive condition except that children received feedback on the performance of their recognition decision exclusively. Consequently, for practice purposes, the characters gave the same examples as in the metacognitive feedback condition, but the examples included performance feedback instead of metacognitive feedback. Feedback for the remaining trials was given visually (green tick for correct recognition, red cross for incorrect recognition) and auditive via headphones. See Table 1 for auditive performance feedback.

#### Active Control Group

The active control group executed six attention control tasks on the tablets. This included three Hearts and Flowers task versions (adapted from Davidson et al., 2006b; Diamond et al., 2007) and three Simon task versions (Simon, 1990). The main difference was that the stimuli in both tasks were exchanged with two animals in each session. Children received brief auditive and visual performance feedback after each incorrect trial. The task was only shortly interrupted by a sound indicating an incorrect answer and a confused smiley face appeared on the screen. However, other than in the metacognitive and performance feedback conditions, children did not indicate confidence judgments. In each session, different stimuli were used to maintain motivation.

### Statistical Analyses

We preregistered our hypotheses and analyses (https://osf.io/mwnsy). We analyzed the data with R (R Core Team, 2021; version: 4.3.0). We imputed missing values with missMDA (version: 1.19) and FactoMineR (version: 2.11) relying on a principal component analysis (Josse & Husson, 2016; Lê et al., 2008). Overall, 5.46% of values were missing and therefore imputed. We tested the intervention effects with linear mixed models (LMM) with lme4 (version: 1.1.36). The dependent variable was uncertainty monitoring computed as the mean difference in confidence judgments between correct and incorrect trials. We set the active control group as the reference group to evaluate the effects of metacognitive and performance feedback. We tested the same model with covariates to account for individual difference variables whose average level might differ across groups. We also preregistered mixed ANOVA to analyze intervention effects. However, we report only LMM because they have multiple advantages compared to ANOVAs: LMM are more powerful, less restrictive regarding model assumptions, more straightforward for interpretation, and can control for nested data structures (Hilbert et al., 2019). Nevertheless, ANOVA results are reported in Supplementary Materials. Data and the R script can be found here: https://osf.io/f3x6k.

## Results

### Descriptives

Compliance with the training sessions was high in all conditions. In the metacognitive feedback group 93%, in the performance feedback group 97%, and in the active control group, 92% attended all sessions. Children with missing training sessions (*n* = 8), missed on average two sessions. They were either ill or had an appointment outside school on the assessment day.

Descriptives revealed positive uncertainty monitoring scores at pre- and posttest across all conditions, indicating that participants reported higher confidence in correct than incorrect memories at pre (*M*_correct_ = 5.13, SD_correct_ = 1.67; *M*_incorrect_ = 4.42, SD_incorrect_ = 1.80)- and posttest (*M*_correct_ = 5.56, SD_correct_ = 1.40; *M*_incorrect_ = 4.83, SD_incorrect_ = 1.57). Paired sample *t*-test confirmed that the confidence differences between correct and incorrect memories were significantly different from zero at pretest *t*(126) = 7.67, *p* < 0.001, and posttest *t*(126) = 6.50, *p* < 0.001.

We compared the groups (metacognitive feedback, performance feedback, active control) with one-way ANOVAs on the dependent variables (memory accuracy and uncertainty monitoring) and potential covariates (age, parental education, inhibition accuracy and reaction time, shifting accuracy and reaction time, working memory, receptive grammar, and fluid intelligence) at pretest. Most importantly, we did not find differences in our primary outcome measures uncertainty monitoring *F* (2, 124) = 0.45, *p* = 0.641 and memory accuracy *F* (2, 124) = 0.89, *p* = 0.414. Moreover, ANOVAs revealed no significant group differences in inhibition accuracy *F* (2, 124) = 0.03, *p* = 0.971; inhibition reaction time *F* (2, 124) = 1.11, *p* = 0.334; shifting accuracy *F* (2, 124) = 0.53, *p* = 0.588; working memory *F* (2, 124) = 0.39, *p* = 0.68; highest parental education *F* (2, 124) = 2.89, *p* = 0.059; and fluid intelligence *F* (2, 124) = 3.02, *p* = 0.052. However, ANOVAs revealed significant group differences in shifting reaction time *F* (2, 124) = 3.92, *p* = 0.022; receptive grammar *F* (2, 124) = 6.01, *p* = 0.003; and age *F* (2, 124) = 3.94, *p* = 0.022. Mean scores are displayed in Table 2. As preregistered, we will include significant group differences at the pretest as covariates in our analyses.

**Table 2:** Means and SD of all variables per condition

	Metacognitive FB	Performance FB	Active Control
*N*	51	33	43
Dependent variables			
Memory accuracy pretest [%]	46.57 (11.68)	43.94 (10.77)	43.77 (11.25)
Memory accuracy posttest [%]	49.02 (19.42)	48.46 (15.7)	43.75 (19.77)
Uncertainty monitoring pretest	0.61 (0.99)	0.72 (1.12)	0.81 (1.03)
Uncertainty monitoring posttest	0.98 (1.19)	0.50 (1.51)	0.59 (1.10)
Covariates			
** Age** [years]	**7.35 (0.45)**	7.39 (0.42)	**7.60 (0.48)**
Parental education	3.12 (0.93)	2.70 (0.85)	2.74 (0.93)
Inhibition accuracy [%]	0.88 (0.17)	0.88 (0.19)	0.87 (0.24)
Inhibition RT [ms]	759.47 (146.27)	713.67 (129.9)	747.88 (139.08)
Shifting accuracy [%]	0.85 (0.06)	0.86 (0.06)	0.84 (0.09)
** Shifting RT [ms]**	**771.32 (117.97)**	**700.49 (119.37)**	732.47 (108.27)
Working memory	6.57 (3.55)	7.02 (3.87)	7.21 (3.53)
** Receptive grammar**	**12.18 (3.77)**	**9.04 (4.66)**	10.61 (3.89)
Fluid intelligence	42.08 (10.35)	39.30 (11.15)	36.33 (12.46)

*Note.* Means and SD in parentheses. *FB *Feedback, *RT* reaction time, Uncertainty Monitoring = Confidence Judgement correct - Confidence Judgement incorrect, significant group differences based on Tukey-corrected pairwise comparisons are in bold

### The Effects of Feedback on Uncertainty Monitoring

We expected uncertainty monitoring to improve more in the metacognitive feedback and performance feedback groups than in the active control group. We fit three LMMs to predict uncertainty monitoring. In model 1, we included condition, time, and the interaction condition*time as fixed effects and a random intercept for the subjects to account for the repeated measures structure of the data. In model 2, we added the covariates (receptive grammar, shifting RT, age) to model 1. In model 3, we added a random intercept for classrooms to model 2. Model 3 allows us to account for the subjects being nested in classrooms. We compared the three models using likelihood ratio tests and the BIC to identify the best-fitting model. Based on these comparisons, we selected model 2, as it significantly improved the fit over model 1 *χ*^2^ (3) = 24.24, *p* < 0.001, did not fit worse than model 3 *χ*^2^ (1) = 0, *p* = 1, and is the most parsimonious model (BIC_Model1_ = 809.31; BIC_Model2_ = 801.68; BIC_Model3_ = 807.21). This indicates that adding covariates enhanced the model’s fit (model 2 vs. model 1), while incorporating the nesting of subjects within classrooms did not provide any additional improvement (model 2 vs. model 3).

**Fig. 3:**
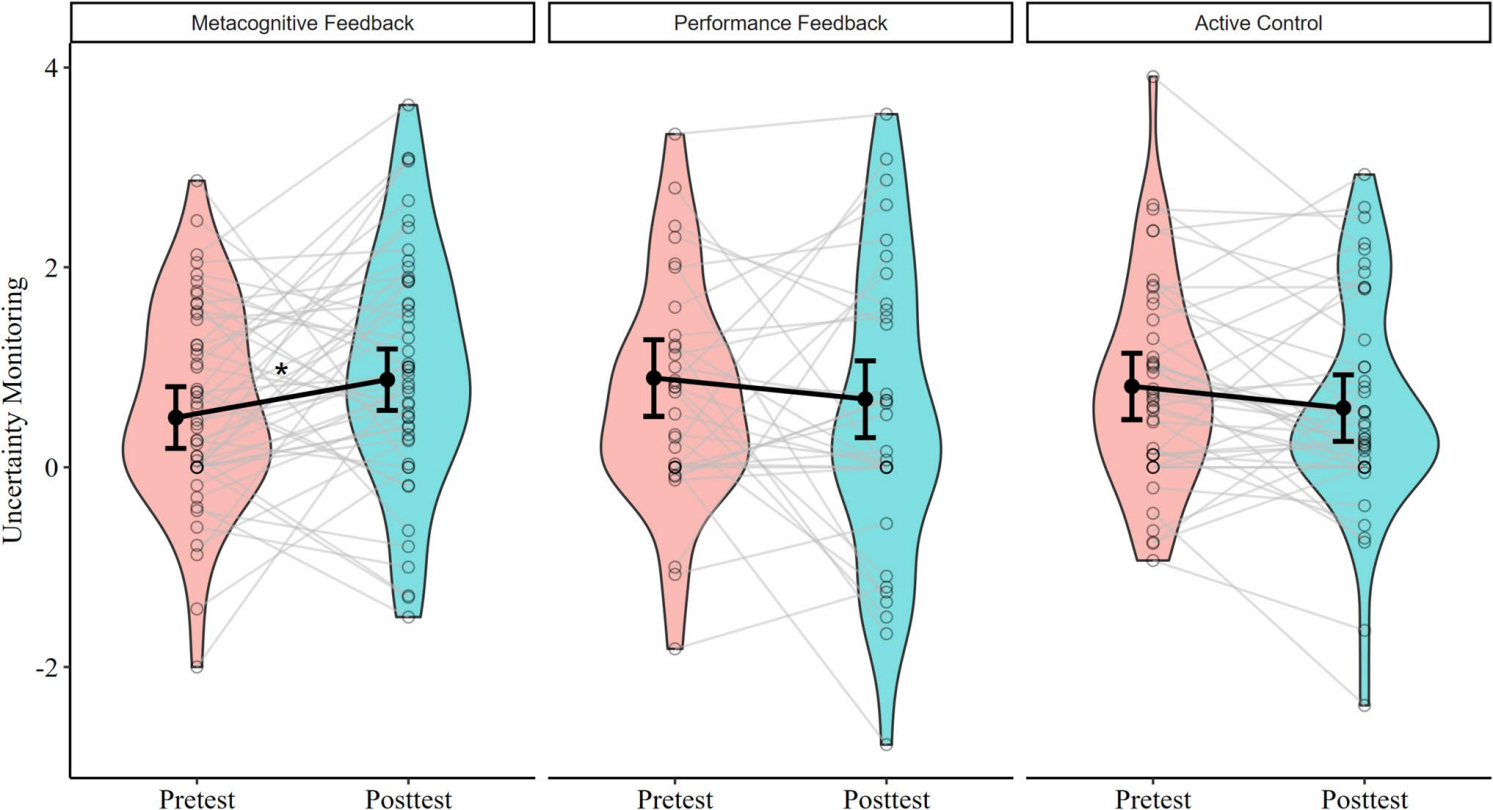
Observed and predicted training effects per condition and time. *Note. *Violin plots depict observed uncertainty monitoring at the pretest (red) and posttest (blue) for each condition (metacognitive feedback, performance feedback, active control). Empty dots represent the observed subject data points at the pretest and posttest, connected by thin grey lines. Black dots indicate the predicted means from the LMM, with 95% confidence intervals. *p<.05 indicates a significant improvement in uncertainty monitoring in the metacognitive feedback group compared to the active control group and the performance feedback group, tested with LMM. Uncertainty monitoring is calculated as confidence judgments on accurate trials – confidence judgments on inaccurate trials

We set the pretest and the active control group as reference groups for contrast analysis. Model 2 revealed that uncertainty monitoring increased from pre- to posttest more in the metacognitive feedback than in the active control (estimate = 0.59). We did not find a difference between the performance feedback and the active control group (estimate = 0). Model 2 is displayed in Table 3. Moreover, we compared the metacognitive group to the performance feedback group by setting the performance feedback group as reference level when computing contrasts in model 2. Contrasts revealed that uncertainty monitoring increased from pretest to posttest more in the metacognitive feedback than in the performance feedback group (estimate = 0.59). In conclusion, LMM suggests that metacognitive feedback, but not performance feedback, benefited children’s uncertainty monitoring (see Fig. 3).

**Table 3:** Linear mixed model predicting uncertainty monitoring

	Estimate	SE	*t*	*p*
*Fixed effects*				
Intercept	− 1.17	1.52	− 0.77	0.442
Performance feedback	0.08	0.26	0.32	0.746
Metacognitive feedback	− 0.31	0.23	− 1.34	0.181
Time	− 0.22	0.19	− 1.12	0.267
Receptive grammar	0.10	0.02	5.00	0
Age	0.01	0.01	0.84	0.403
Shifting RT	0	0	− 0.39	0.696
Time × performance FB	0	0.29	0.02	0.987
** Time × metacognitive FB**	0.59	0.26	2.26	**0.026**
*Random effects*	*Variance*		*SD*	
Subject (intercept)	0.38		0.61	
Residual	0.81		0.90	

*Note. SE =* Standard error, *t* = t-value, *FB* Feedback, *SD* Standard Deviation, Pretest and the active control group are set as reference levels, significant terms at *p<*.05 are in bold.

### The Effects of Feedback on Memory Accuracy

Our exploratory analyses involved testing for training effects on memory accuracy. To enable comparison with effects on uncertainty monitoring, we relied on the same LMM as described previously (model 2). We predicted memory accuracy by including condition, time, the interaction condition*time, and the covariates (receptive grammar, shifting RT, age) as fixed effects, and a random intercept for subjects to account for the repeated measure structure of the data. We set the pretest and the active control group as reference groups for contrast analysis. As can be seen in Table 4, metacognitive feedback (estimate = 0.02) and performance feedback (estimate = 0.05) did not affect memory accuracy.

**Table 4:** Linear Mixed Model predicting memory accuracy

	Estimate	SE	*t*	*p*
*Fixed effects*				
Intercept	0.17	0.21	0.85	0.399
Performance feedback	0.02	0.04	0.61	0.543
Metacognitive feedback	0.02	0.03	0.63	0.531
Time	0	0.03	− 0.01	0.995
Receptive grammar	0.01	0	3.81	0
Age	0	0	1.04	0.301
Shifting RT	0	0	− 0.51	0.612
Time × performance FB	0.05	0.04	1.09	0.276
Time × metacognitive FB	0.02	0.04	0.67	0.507
*Random effects*	*Variance*		*SD*	
Subject (intercept)	0.01		0.08	
Residual	0.02		0.13	

*Note. SE* = Standard error, *t* t-value, *FB* Feedback, *SD* Standard Deviation, Pretest and the active control group are set as reference levels

## Discussion

The overarching goal of the present study was to evaluate the effects of a training comprising six computerized sessions, including metacognitive or performance feedback, on primary school children’s uncertainty monitoring. We randomly assigned classrooms to either metacognitive feedback, performance feedback, or an active control group. We found that metacognitive feedback, but not performance feedback, increased uncertainty monitoring. However, neither metacognitive nor performance feedback increased memory accuracy. In the following paragraphs, we will discuss our results and propose suggestions for future training studies.

Metacognitive feedback improved primary school children’s uncertainty monitoring (see Fig. 3). This is in line with previous studies suggesting metacognitive feedback benefits of uncertainty monitoring in 4- to 10-year-olds (Geurten & Meulemans, 2017; Oudman et al., 2022; van Loon & Roebers, 2020) and adults (Callender et al., 2016; Miller & Geraci, 2011; Nietfeld et al., 2006). However, our results must be interpreted cautiously as we found effects when relying on LMM (Table 3) but not with mixed ANOVAs (reported in the Supplementary Materials). LMM are more powerful and less restrictive regarding model assumptions than ANOVAs and are, therefore, more sensitive to detecting small effects in intervention designs (Hilbert et al., 2019). Our findings point to a small benefit of metacognitive feedback for children’s uncertainty monitoring, consistent with previous training studies. Van Loon and Roebers (2020) found that metacognitive feedback increases error detection in 6-year-olds, but two-thirds of the errors were still not recognized. Also, Oudman et al. (2022) found that metacognitive feedback can reduce overconfidence but that 10-year-olds will likely remain overconfident. This might indicate that improving children’s monitoring accuracy requires multiple (many more) and consistent repetitions. Future interventions should investigate whether more training sessions lead to higher monitoring accuracy and whether, more generally, there is a dose effect in the relationship between the extent of feedback sessions and improvements in uncertainty monitoring.

The performance feedback condition did not yield any improvements in uncertainty monitoring, with this finding being similar to previous research with 4- to 6-year-olds (Lipko et al., 2009, 2012; O’Leary & Sloutsky, 2017; Xia et al., 2022). However, some studies with children as young as 5 reported performance feedback benefits for children’s uncertainty monitoring (van Loon & Roebers, 2020; van Loon et al., 2017). Van Loon et al. (2017) gave children performance feedback before they indicated their uncertainty, and the content of the feedback remained visible during children’s monitoring judgments. This procedure arguably reduced processing demands and obviously confronted children with their errors. Thus, it may be particularly demanding for children to identify the cues to accuracy if the task also requires that children keep feedback in mind. This difficulty may be accentuated by the fact that we provided performance feedback only after children indicated their uncertainty. Our elected procedure required children to link the feedback retrospectively to memory decisions, and their typically very high confidence judgment may have interfered. Finally, performance feedback might be easier to relate to uncertainty for older children. Studies with 9- and 10-year-olds found benefits of performance feedback for uncertainty monitoring (Oudman et al., 2022; van Loon & Roebers, 2017). Performance feedback alone might not sufficiently scaffold young children’s recognition and reliance on the most informative cues for uncertainty. In sum, the benefits of performance feedback might depend on the children’s age, the timing of performance feedback (before or after the uncertainty judgment), and the interaction of age and timing. It remains up to future research to clarify the most promising approach to improve children’s uncertainty monitoring with performance feedback.

Metacognitive and performance feedback did not increase memory accuracy. This finding is inconsistent with previous research with 5- and 6-year-olds suggesting that performance feedback leads to higher accuracy on related, subsequent cognitive tasks (Muis et al., 2015; van Loon & Roebers, 2020), but in these studies, the training and the tests utilized the same task. In contrast, we investigated how performance feedback during the intervention session transferred to a subsequent task on which feedback was never provided. Young children might need immediate performance feedback to increase their accuracy. Moreover, our study design did not provide many opportunities for participants to initiate self-regulated behavior based on their monitoring. For instance, already 3-year-olds rely on uncertainty monitoring to control learning processes, such as help-seeking and withdrawing incorrect answers (Roebers, 2017), which is crucial for accuracy (Destan et al., 2014; Ghetti et al., 2013; Hembacher & Ghetti, 2013). In the present study, study time was predetermined, participants could not ask for help, and answers could not be withdrawn. Investigating how feedback affects metacognitive control may be an interesting question for future research.

Following Efklides’ (2008) multifaceted and multilevel model of metacognition, interventions targeting the social level of metacognition might be more promising. For our training sessions, we pre-recorded auditive feedback, which was then delivered via headphones. Computerized feedback might be different from face-to-face feedback by a social agent. For instance, a social agent can verify that a child understands the feedback and it might be more motivating to receive feedback from a social agent than from a recorded voice. Future research should investigate whether feedback by a social agent is more effective than recorded feedback. Indeed, a recent study found that disagreement with an adult expert reduces overconfidence between 4- and 6-year-olds (Langenhoff et al., 2024). Alternatively, we should explore whether an embedded token system (get points for the correspondence between recall and monitoring accuracy and get a reward for points, e.g., access to a funny game in the end) within and across training sessions could compensate for the absence of a social partner, maintaining the advantage of a resource-sparing learning application (e.g., Cogmed ®, Deniz Aksayli et al., 2019).

We acknowledge the circumscribed limitations of the present study. At pretest, participants in our experimental conditions unexpectedly differed in receptive grammar, shifting reaction time, and age. Therefore, we included these variables as covariates in our model. However, it is crucial to note that we did not find baseline differences in our primary outcome variables, uncertainty monitoring and memory accuracy, as well as in important control variables (highest parental education, inhibition accuracy and RT, shifting accuracy, working memory, and fluid intelligence). Moreover, our pre- and post-test may have been a bit difficult for the investigated age group, as indicated by the number of participants scoring below the chance level (*n* = 46). For future studies, we recommend carefully considering task difficulty when choosing the stimuli, such as the similarity, the presentation time, and the number of to-be-learned stimuli.

Despite the limitations that will be informative for future studies, the present study has several strengths. This is one of the first studies to investigate the effects of feedback during repeated training sessions on children’s uncertainty monitoring tested on a different task. Moreover, the present study examines how training effects transfer to a task without feedback. The benefits of metacognitive feedback for uncertainty monitoring are especially noteworthy as both the recognition task and the confidence scale used in the pre- and posttest differed from the training sessions. This allows for estimating the robustness of feedback effects for children’s uncertainty monitoring. Including an active control group, we account for placebo effects (Green et al., 2019).

### Conclusion

This is one of the first studies investigating the potential benefits of systematic and repeated metacognitive and performance feedback for children’s uncertainty monitoring. Metacognitive feedback, but not performance feedback, improved children’s uncertainty monitoring. However, the effects were small, and metacognitive and performance feedback did not increase memory accuracy. Perhaps, more training sessions, including more items, are necessary for young children to translate their improved uncertainty monitoring into effective recognition decisions. The number of required sessions and items per session is one of the most important aspects to be clarified in future studies. In our view, this line of research should be pursued as the development of suitable and efficient learning apps is of great importance in educational settings, for example, to compensate for large classrooms and limited teachers who cannot always provide personal feedback to each student.

## Supplementary Information

Below is the link to the electronic supplementary material.

ESM 1 (DOCX 16.2 KB)

## Data Availability

Data and R scripts can be retrieved here: https://osf.io/f3x6k

## References

[CR1] Bayard, N. S., van Loon, M. H., Steiner, M., & Roebers, C. M. (2021). Developmental improvements and persisting difficulties in children’s metacognitive monitoring and control skills: Cross-sectional and longitudinal perspectives. *Child Development,**92*(3), 1118–1136. 10.1111/cdev.13486 33529372 10.1111/cdev.13486 PMC8248442

[CR2] Buehler, F. J., Orth, U., Krauss, S., & Roebers, C. M. (2025). Language abilities and metacognitive monitoring development: Divergent longitudinal pathways for native and non-native speaking children. *Learning and Instruction*, *102043*(95). 10.1016/j.learninstruc.2024.102043

[CR3] Callender, A. A., Franco-Watkins, A. M., & Roberts, A. S. (2016). Improving metacognition in the classroom through instruction, training, and feedback. *Metacognition and Learning,**11*(2), 215–235. 10.1007/s11409-015-9142-6

[CR4] Camerota, M., Willoughby, M. T., & Blair, C. B. (2019). Speed and accuracy on the hearts and flowers task interact to predict child outcomes. *Psychological Assessment* , *31*(8), 995–1005. 10.1037/pas0000725 10.1037/pas0000725 PMC6675624 31033313

[CR5] Carr, M., Kurtz, B. E., Schneider, W., Turner, L. A., & Borkowski, J. G. (1989). Strategy acquisition and transfer among American and German children: Environmental influences on metacognitive development. *Developmental Psychology,**25*(5), 765–771. 10.1037/0012-1649.25.5.765

[CR6] Clerc, J., Miller, P. H., & Cosnefroy, L. (2014). Young children’s transfer of strategies: Utilization deficiencies, executive function, and metacognition. *Developmental Review,**34*(4), 378–393. 10.1016/j.dr.2014.10.002

[CR7] Cornoldi, C., Carretti, B., Drusi, S., & Tencati, C. (2015). Improving problem solving in primary school students: The effect of a training programme focusing on metacognition and working memory. *British Journal of Educational Psychology,**85*(3), 424–439. 10.1111/bjep.12083 26099785 10.1111/bjep.12083

[CR8] Corsi, P. M. (1972). *Memory and the medial temporal region of the brain* [unpublished doctoral thesis]. McGill University.

[CR9] Davidson, M. C., Amso, D., Anderson, L. C., & Diamond, A. (2006). Development of cognitive control and executive functions from 4 to 13 years: Evidence from manipulations of memory, inhibition, and task switching. *Neuropsychologia,**44*(11), 2037–2078. 10.1016/J.NEUROPSYCHOLOGIA.2006.02.006 16580701 10.1016/j.neuropsychologia.2006.02.006 PMC1513793

[CR10] Deniz Aksayli, N., Sala, G., & Gobet, F. (2019). The cognitive and academic benefits of Cogmed: A meta-analysis. *Educational Research Review,**27*, 229–243. 10.1016/j.edurev.2019.04.003

[CR11] Destan, N., Hembacher, E., Ghetti, S., & Roebers, C. M. (2014). Early metacognitive abilities: The interplay of monitoring and control processes in 5- to 7-year-old children. *Journal of Experimental Child Psychology,**126*, 213–228. 10.1016/j.jecp.2014.04.001 24945686 10.1016/j.jecp.2014.04.001

[CR12] Destan, N., & Roebers, C. M. (2015). What are the metacognitive costs of young children’s overconfidence? *Metacognition and Learning,**10*(3), 347–374. 10.1007/s11409-014-9133-z

[CR13] Diamond, A., Barnett, W. S., Thomas, J., & Munro, S. (2007). Preschool program improves cognitive control. *Science,**318*(5855), 1387–1388. 10.1126/science.1151148 18048670 10.1126/science.1151148 PMC2174918

[CR14] Dunlosky, J., & Metcalfe, J. (2009). *Metacognition: A textbook for cognitive, educational, life span & applied psychology*. Sage Publications.

[CR15] Dunlosky, J., Mueller, M. L., spsampsps Thiede, K. W. (2016). Methodology for investigating human metamemory: Problems and pitfalls. In J. Dunlosky spsampsps S. K. Tauber (Eds.), *The Oxford handbook of metamemory*. Oxford University Press. 10.1093/oxfordhb/9780199336746.013.14

[CR16] Dunlosky, J., & Thiede, K. W. (2013). Four cornerstones of calibration research: Why understanding students’ judgments can improve their achievement. *Learning and Instruction,**24*(1), 58–61. 10.1016/j.learninstruc.2012.05.002

[CR17] Efklides, A. (2008). Metacognition - Defining its facets and levels of functioning in relation to self-regulation and co-regulation. *European Psychologist,**13*(4), 277–287. 10.1027/1016-9040.13.4.277

[CR18] Efklides, A., & Metallidou, P. (2020). Applying metacognition and self-regulated learning in the classroom. *In Oxford Research Encyclopedia of Education*. 10.1093/acrefore/9780190264093.013.961

[CR19] Fandakova, Y., Selmeczy, D., Leckey, S., Grimm, K. J., Wendelken, C., Bunge, S. A., & Ghetti, S. (2017). Changes in ventromedial prefrontal and insular cortex support the development of metamemory from childhood into adolescence. *Proceedings of the National Academy of Sciences of the United States of America,**114*(29), 7582–7587. 10.1073/pnas.1703079114 28673976 10.1073/pnas.1703079114 PMC5530674

[CR20] Faul, F., Erdfelder, E., Lang, A.-G., & Buchner, A. (2007). G*Power 3: A flexible statistical power analysis program for the social, behavioral, and biomedical sciences. *Behavior Research Methods,**39*(2), 175–191. 17695343 10.3758/bf03193146

[CR21] Federal Statistical Office. (2021). *Höchste abgeschlossene Ausbildung, nach Migrationsstatus, verschiedenen soziodemografischen Merkmalen und Grossregion [Highest level of education, by migration status, various sociodemographic characteristics and region]*. https://www.bfs.admin.ch/bfs/en/home/statistics/population/migration-integration/integration-indicators/indicators/highest-educational-level.assetdetail.20164022.html

[CR22] Finn, B., & Metcalfe, J. (2014). Overconfidence in children’s multi-trial judgments of learning. *Learning and Instruction,**32*, 1–9. 10.1016/j.learninstruc.2014.01.001

[CR23] Fox-Boyer, A. V. (2011). *TROG-D. Test zu Überprüfung des Grammatikverständnisses [Grammar comprehension test]*. Idstein Schulz-Kirchner Verlag.

[CR24] Freeman, E. E., Karayanidis, F., & Chalmers, K. A. (2017). Metacognitive monitoring of working memory performance and its relationship to academic achievement in Grade 4 children. *Learning and Individual Differences,**57*, 58–64. 10.1016/j.lindif.2017.06.003

[CR25] Frick, A., & Möhring, W. (2016). A matter of balance: Motor control is related to children’s spatial and proportional reasoning skills. *Frontiers in Psychology,**6*(2049), 1–10. 10.3389/fpsyg.2015.02049 10.3389/fpsyg.2015.02049 PMC4709580 26793157

[CR26] Geurten, M., & Meulemans, T. (2017). The effect of feedback on children’s metacognitive judgments: A heuristic account. *Journal of Cognitive Psychology,**29*(2), 184–201. 10.1080/20445911.2016.1229669

[CR27] Geurten, M., & Willems, S. (2016). Metacognition in early childhood: Fertile ground to understand memory development? *Child Development Perspectives,**10*(4), 263–268. 10.1111/CDEP.12201

[CR28] Ghetti, S., Hembacher, E., & Coughlin, C. A. (2013). Feeling uncertain and acting on it during the preschool years: A metacognitive approach. *Child Development Perspectives,**7*(3), 160–165. 10.1111/cdep.12035

[CR29] Gonzales, C. R., Merculief, A., McClelland, M. M., & Ghetti, S. (2022). The development of uncertainty monitoring during kindergarten: Change and longitudinal relations with executive function and vocabulary in children from low-income backgrounds. *Child Development,**93*(2), 1–16. 10.1111/cdev.13714 10.1111/cdev.13714 34889459

[CR30] Green, S. C., Bavelier, D., Kramer, A. F., Vinogradov, S., Ansorge, U., Ball, K. K., Bingel, U., Chein, J. M., Colzato, L. S., Edwards, J. D., Facoetti, A., Gazzaley, A., Gathercole, S. E., Ghisletta, P., Gori, S., Granic, I., Hillman, C. H., Hommel, B., Jaeggi, S. M., … Witt, C. M. (2019). Improving methodological standards in behavioral interventions for cognitive enhancement. *Journal of Cognitive Enhancement*, *3*(1), 2–29. 10.1007/s41465-018-0115-y

[CR31] Haddara, N., & Rahnev, D. (2022). The impact of feedback on perceptual decision-making and metacognition: Reduction in bias but no change in sensitivity. *Psychological Science,**33*(2), 259–275. 10.1177/09567976211032887 35100069 10.1177/09567976211032887 PMC9096460

[CR32] Hagmann-von Arx, P., & Grob, A. (2014). *RIAS. Reynolds Intellectual Assessment Scales and Screening. Deutschsprachige Adaptation der Reynolds Intellectual Assessment Scales (RIAS) & des Reynolds Intellectual Screening Test (RIST) von Cecil R. Reynolds und Randy W. Kamphaus [German version]*. Huber.

[CR33] Hembacher, E., & Ghetti, S. (2013). How to bet on a memory: Developmental linkages between subjective recollection and decision making. *Journal of Experimental Child Psychology,**115*(3), 436–452. 10.1016/j.jecp.2013.03.010 23665179 10.1016/j.jecp.2013.03.010

[CR34] Hembacher, E., & Ghetti, S. (2014). Don’t look at my answer: Subjective uncertainty underlies preschoolers’ exclusion of their least accurate memories. *Psychological Science,**25*(9), 1768–1776. 10.1177/0956797614542273 25015686 10.1177/0956797614542273

[CR35] Hilbert, S., Stadler, M., Lindl, A., Naumann, F., & Bühner, M. (2019). Analyzing longitudinal intervention studies with linear mixed models. *TPM,**26*(1), 101–119. 10.4473/TPM26.1.6

[CR36] Josse, J., & Husson, F. (2016). missMDA: A package for handling missing values in multivariate data analysis. *Journal of Statistical Software*, *70*, 1–31. 10.18637/JSS.V070.I01

[CR37] Koriat, A., & Ackerman, R. (2010). Choice latency as a cue for children’s subjective confidence in the correctness of their answers. *Developmental Science,**13*(3), 441–453. 10.1111/j.1467-7687.2009.00907.x 20443965 10.1111/j.1467-7687.2009.00907.x

[CR38] Koriat, A., spsampsps Shitzer-Reichert, R. (2002). Metacognitive judgments and their accuracy. In P. Chambres, M. Izaute, spsampsps P.-J. Marescaux (Eds.), *Metacognition: Process, function and use* (pp. 1–17). Springer. 10.1007/978-1-4615-1099-4_1

[CR39] Krebs, S. S., & Roebers, C. M. (2012). The impact of retrieval processes, age, general achievement level, and test scoring scheme for children’s metacognitive monitoring and controlling. *Metacognition and Learning,**7*(2), 75–90. 10.1007/s11409-011-9079-3

[CR40] Langenhoff, A. F., Srinivasan, M., & Engelmann, J. M. (2024). Disagreement reduces overconfidence and prompts exploration in young children. *Child Development*. 10.1111/cdev.14098 38588018 10.1111/cdev.14098

[CR41] Lê, S., Josse, J., & Husson, F. (2008). FactoMineR: An R package for multivariate analysis. *Journal of Statistical Software*, *25*(1), 1–18. 10.18637/JSS.V025.I01

[CR42] Lipko, A. R., Dunlosky, J., Lipowski, S. L., & Merriman, W. E. (2012). Young children are not underconfident with practice: The benefit of ignoring a fallible memory heuristic. *Journal of Cognition and Development,**13*(2), 174–188. 10.1080/15248372.2011.577760

[CR43] Lipko, A. R., Dunlosky, J., & Merriman, W. E. (2009). Persistent overconfidence despite practice: The role of task experience in preschoolers’ recall predictions. *Journal of Experimental Child Psychology,**103*(2), 152–166. 10.1016/j.jecp.2008.10.002 19058813 10.1016/j.jecp.2008.10.002

[CR44] Macoun, S. J., Pyne, S., MacSween, J., Lewis, J., & Sheehan, J. (2022). Feasibility and potential benefits of an attention and executive function intervention on metacognition in a mixed pediatric sample. *Applied Neuropsychology: Child,**11*(3), 240–252. 10.1080/21622965.2020.1794867 32701379 10.1080/21622965.2020.1794867

[CR45] Maurer, M. N., & Roebers, C. M. (2021). New insights into visual-motor integration exploring process measures during copying shapes. *Psychology of Sport and Exercise,**55*(101954), 1–9. 10.1016/j.psychsport.2021.101954

[CR46] Miller, T. M., & Geraci, L. (2011). Training metacognition in the classroom: The influence of incentives and feedback on exam predictions. *Metacognition and Learning,**6*(3), 303–314. 10.1007/s11409-011-9083-7

[CR47] Muis, K. R., Ranellucci, J., Trevors, G., & Duffy, M. C. (2015). The effects of technology-mediated immediate feedback on kindergarten students’ attitudes, emotions, engagement and learning outcomes during literacy skills development. *Learning and Instruction,**38*, 1–13. 10.1016/j.learninstruc.2015.02.001

[CR48] Nelson, T. O., & Narens, L. (1990). Metamemory: A theoretical framework and new findings. *Psychology of Learning and Motivation - Advances in Research and Theory,**26*, 125–173.

[CR49] Nietfeld, J. L., Cao, L., & Osborne, J. W. (2006). The effect of distributed monitoring exercises and feedback on performance, monitoring accuracy, and self-efficacy. *Metacognition and Learning,**1*(2), 159–179. 10.1007/s10409-006-9595-6

[CR50] O’Leary, A. P., & Sloutsky, V. M. (2017). Carving metacognition at its joints: Protracted development of component processes. *Child Development,**88*(3), 1015–1032. 10.1111/cdev.12644 27759890 10.1111/cdev.12644 PMC5397377

[CR51] Oudman, S., van de Pol, J., & van Gog, T. (2022). Effects of self-scoring their math problem solutions on primary school students’ monitoring and regulation. *Metacognition and Learning,**17*(1), 213–239. 10.1007/s11409-021-09281-9

[CR52] R Core Team. (2021). *R: A language and environment for statistical computing [Computer software]*. https://www.r-project.org/

[CR53] Raaijmakers, S. F., Baars, M., Paas, F., Van Merriënboer, J. J. G., & Van Gog, T. (2019). Effects of self-assessment feedback on self-assessment and task-selection accuracy. *Metacognition and Learning,**14*, 21–42. 10.1007/s11409-019-09189-5

[CR54] Reynolds, C. R., & Kamphaus, R. W. (2003). *RIAS. Reynolds Intellectual Assessment Scales*. PAR.

[CR55] Roebers, C. M. (2002). Confidence judgments in children’s and adults’ event recall and suggestibility. *Developmental Psychology,**38*(6), 1052–1067. 10.1037/0012-1649.38.6.1052 12428714 10.1037//0012-1649.38.6.1052

[CR56] Roebers, C. M. (2017). Executive function and metacognition: Towards a unifying framework of cognitive self-regulation. *Developmental Review,**45*, 31–51. 10.1016/j.dr.2017.04.001

[CR57] Roebers, C. M. (2022). Six- to eight-year-olds’ performance in the Heart and Flower task: Emerging proactive cognitive control. *Frontiers in Psychology,**13*(August), 1–14. 10.3389/fpsyg.2022.923615 10.3389/fpsyg.2022.923615 PMC9404302 36033019

[CR58] Schraw, G. (2009). A conceptual analysis of five measures of metacognitive monitoring. *Metacognition and Learning,**4*, 33–45. 10.1007/s11409-008-9031-3

[CR59] Schraw, G., Crippen, K. J., & Hartley, K. (2006). Promoting self-regulation in science education: Metacognition as part of a broader perspective on learning. *Research in Science Education,**36*, 111–139. 10.1007/s11165-005-3917-8

[CR60] Simon, J. R. (1990). The effects of an irrelevant directional cue on human information processing. *Advances in Psychology,**65*, 31–86. 10.1016/S0166-4115(08)61218-2

[CR61] Takacs, Z. K., & Kassai, R. (2019). The efficacy of different interventions to foster children’s executive function skills: A series of meta-analyses. *Psychological Bulletin*, *145*(7), 653–697. 10.1037/bul0000195 10.1037/bul0000195 31033315

[CR62] van Loon, M. H., Destan, N., Spiess, M. A., de Bruin, A., & Roebers, C. M. (2017). Developmental progression in performance evaluations: Effects of children’s cue-utilization and self-protection. *Learning and Instruction,**51*, 47–60. 10.1016/j.learninstruc.2016.11.011

[CR63] van Loon, M. H., & Roebers, C. M. (2017). Effects of feedback on self-evaluations and self-regulation in elementary school. *Applied Cognitive Psychology,**31*(5), 508–519. 10.1002/acp.3347

[CR64] van Loon, M. H., & Roebers, C. M. (2020). Using feedback to improve monitoring judgment accuracy in kindergarten children. *Early Childhood Research Quarterly,**53*, 301–313. 10.1016/j.ecresq.2020.05.007

[CR65] van Loon, M. H., spsampsps Roebers, C. M. (2021). Using feedback to support children when monitoring and controlling their learning. In D. Moraitou spsampsps P. Metallidou (Eds.), *Trends and prospects in metacognition research across the life span. A tribute to Anastasia Efklides.* (pp. 161–184). Springer. 10.1007/978-3-030-51673-4_8

[CR66] Xia, M., Poorthuis, A. M. G., Zhou, Q., & Thomaes, S. (2022). Young children’s overestimation of performance: A cross-cultural comparison. *Child Development,**93*(2), e207–e221. 10.1111/cdev.13709 34741531 10.1111/cdev.13709 PMC9298085

