# Supplementary material for: Repeated Feedback Can Benefit Seven-Year-old’s Uncertainty Monitoring in a Memory Task
Source: J Cogn Enhanc. 2025 Apr 3;9(2):230–43. doi: 10.1007/s41465-025-00322-8 (PMC12122647; doi:10.1007/s41465-025-00322-8)
Supplement: Supplementary file 1 — (DOCX 16.2 KB) [file 41465_2025_322_MOESM1_ESM.docx]

**Supplementary Materials**

We tested a 2 (Time: pre- vs. posttraining) x 3 (Condition: metacognitive feedback vs. performance feedback vs. active control) mixed ANOVA with time as a within-person variable and condition as a between-person variable. The dependent variable was uncertainty monitoring. The mixed ANOVA revealed no main effects of time *F* (1, 124) = 0.021, *p* = .885, $\text{η}_{p}^{2}$= .00 or group *F*(2, 124) = 0.39, *p* = .681, $\text{η}_{p}^{2}$= .01 but a significant time*condition interaction, *F* (2, 124) = 3.31, *p* = .04, $\text{η}_{p}^{2}$= .05. We followed up on the interaction with three univariate ANOVAs, testing the effect from pre- to posttest separately for the metacognitive feedback, performance feedback, and active control groups. Results revealed no significant difference from pre- to posttest for the metacognitive feedback group *F* (1, 50) = 4.17, *p* = .138, $\text{η}_{p}^{2}$= .08; the performance feedback *F*(1, 32) = 0.60, *p* = .443, $\text{η}_{p}^{2}$= .02; or the active control group *F* (1, 42) = 2.42, *p* = .254, $\text{η}_{p}^{2}$= .05. All reported p-values were adjusted using Holm-Bonferroni correction for multiple comparisons.

We conducted the same model with covariates to account for group differences at the pretest. The covariate receptive grammar was significantly related to uncertainty monitoring *F* (1, 121) = 25.00, *p* < .001, $\text{η}_{p}^{2}$= .17, however shifting reaction time *F* (1, 121) = 0.15, *p* = .696, $\text{η}_{p}^{2}$= .00 and age *F* (1, 121) = 0.70, *p* = .43, $\text{η}_{p}^{2}$= .01 were not related to uncertainty monitoring. The mixed ANCOVA revealed no main effects of time *F* (1, 121) = 2.70, *p* = .13, $\text{η}_{p}^{2}$= .02 or group *F* (2, 121) = 0.13, *p* = .882, $\text{η}_{p}^{2}$= .00 but – again - a significant time*condition interaction *F* (2, 121) = 3.94, *p* = .022, $\text{η}_{p}^{2}$= .06. We followed up on the interaction with three univariate ANCOVAs, testing the effect from pre- to posttest separately for the metacognitive feedback, performance feedback, and active control groups, when controlling for the covariates. Results revealed no significant difference from pre- to posttest for the metacognitive feedback group *F* (1, 47) = 4.42, *p* = .123; $\text{η}_{p}^{2}$= .09 the performance feedback *F* (1, 29) = 0.592, *p* = .448; $\text{η}_{p}^{2}$= .02 or the active control group *F* (1, 39) = 2.74, *p* = .212, $\text{η}_{p}^{2}$= .07. All reported p-values were adjusted using Holm-Bonferroni correction for multiple comparisons.
